# Supplementary material for: Multi‐dataset identification of innovative feature genes and molecular mechanisms in keratoconus
Source: J Cell Mol Med. 2024 Sep 19;28(18):e70079. doi: 10.1111/jcmm.70079 (PMC11412914; doi:10.1111/jcmm.70079)
Supplement: Supplementary file 2 — Material S2. [file JCMM-28-e70079-s002.zip › 7.GSEA analysis/3.DBNDD1/KEGG_OXIDATIVE_PHOSPHORYLATION.html]

Details for gene set KEGG\_OXIDATIVE\_PHOSPHORYLATION[GSEA]

|  || Dataset | DBNDD1.group.cls#h\_versus\_l |
| Phenotype | group.cls#h\_versus\_l |
| Upregulated in class | h |
| GeneSet | KEGG\_OXIDATIVE\_PHOSPHORYLATION |
| Enrichment Score (ES) | 0.16832227 |
| Normalized Enrichment Score (NES) | 0.72309035 |
| Nominal p-value | 0.7755511 |
| FDR q-value | 1.0 |
| FWER p-Value | 1.0 |
Table: GSEA Results Summary

  

Fig 1: Enrichment plot: KEGG\_OXIDATIVE\_PHOSPHORYLATION      
 Profile of the Running ES Score & Positions of GeneSet Members on the Rank Ordered List

  

| SYMBOL | TITLE | RANK IN GENE LIST | RANK METRIC SCORE | RUNNING ES | CORE ENRICHMENT || 1 | ATP4B | na | 3217 | 0.243 | -0.0214 | Yes |
| 2 | ATP6V0D2 | na | 4031 | 0.213 | 0.0177 | Yes |
| 3 | COX7A2L | na | 8857 | 0.088 | -0.0957 | Yes |
| 4 | COX6A2 | na | 8938 | 0.087 | -0.0725 | Yes |
| 5 | COX4I2 | na | 9035 | 0.085 | -0.0503 | Yes |
| 6 | ATP6V1B2 | na | 9429 | 0.079 | -0.0382 | Yes |
| 7 | PPA2 | na | 9939 | 0.072 | -0.0317 | Yes |
| 8 | COX6B2 | na | 10294 | 0.067 | -0.0221 | Yes |
| 9 | NDUFB5 | na | 10463 | 0.065 | -0.0078 | Yes |
| 10 | ATP6V0A4 | na | 11197 | 0.056 | -0.0125 | Yes |
| 11 | NDUFC2 | na | 11325 | 0.054 | -0.0002 | Yes |
| 12 | NDUFV3 | na | 11702 | 0.050 | 0.0037 | Yes |
| 13 | COX15 | na | 11823 | 0.049 | 0.0146 | Yes |
| 14 | ATP6V0B | na | 11849 | 0.049 | 0.0282 | Yes |
| 15 | ATP6V0E1 | na | 12510 | 0.042 | 0.0216 | Yes |
| 16 | NDUFAB1 | na | 12930 | 0.039 | 0.0209 | Yes |
| 17 | NDUFB1 | na | 13049 | 0.038 | 0.0286 | Yes |
| 18 | NDUFS6 | na | 13068 | 0.038 | 0.0392 | Yes |
| 19 | NDUFB10 | na | 13393 | 0.035 | 0.0402 | Yes |
| 20 | UQCR11 | na | 13449 | 0.035 | 0.0488 | Yes |
| 21 | NDUFB8 | na | 13532 | 0.034 | 0.0564 | Yes |
| 22 | NDUFB7 | na | 13785 | 0.032 | 0.0584 | Yes |
| 23 | COX8A | na | 13890 | 0.031 | 0.0645 | Yes |
| 24 | SDHC | na | 14109 | 0.029 | 0.0668 | Yes |
| 25 | NDUFA11 | na | 14162 | 0.029 | 0.0738 | Yes |
| 26 | NDUFA9 | na | 14400 | 0.027 | 0.0749 | Yes |
| 27 | NDUFA4L2 | na | 14523 | 0.026 | 0.0792 | Yes |
| 28 | NDUFS1 | na | 14531 | 0.026 | 0.0867 | Yes |
| 29 | ATP6AP1 | na | 14609 | 0.026 | 0.0920 | Yes |
| 30 | ATP6V0E2 | na | 14809 | 0.024 | 0.0934 | Yes |
| 31 | NDUFS4 | na | 14861 | 0.024 | 0.0989 | Yes |
| 32 | NDUFA10 | na | 14952 | 0.023 | 0.1032 | Yes |
| 33 | NDUFS2 | na | 15047 | 0.022 | 0.1071 | Yes |
| 34 | COX6B1 | na | 15292 | 0.021 | 0.1061 | Yes |
| 35 | COX5B | na | 15394 | 0.020 | 0.1091 | Yes |
| 36 | COX11 | na | 15441 | 0.020 | 0.1135 | Yes |
| 37 | COX7B2 | na | 15452 | 0.020 | 0.1190 | Yes |
| 38 | COX7B | na | 15536 | 0.019 | 0.1222 | Yes |
| 39 | NDUFS8 | na | 15627 | 0.018 | 0.1250 | Yes |
| 40 | COX7A2 | na | 15716 | 0.018 | 0.1277 | Yes |
| 41 | ATP6V1G3 | na | 15776 | 0.017 | 0.1311 | Yes |
| 42 | NDUFA1 | na | 15785 | 0.017 | 0.1359 | Yes |
| 43 | NDUFC1 | na | 15837 | 0.017 | 0.1395 | Yes |
| 44 | NDUFA7 | na | 15872 | 0.017 | 0.1434 | Yes |
| 45 | UQCR10 | na | 16010 | 0.016 | 0.1441 | Yes |
| 46 | NDUFB3 | na | 16011 | 0.016 | 0.1487 | Yes |
| 47 | SDHD | na | 16304 | 0.014 | 0.1444 | Yes |
| 48 | NDUFA8 | na | 16485 | 0.013 | 0.1429 | Yes |
| 49 | NDUFB2 | na | 16493 | 0.013 | 0.1464 | Yes |
| 50 | LHPP | na | 16559 | 0.012 | 0.1482 | Yes |
| 51 | UQCRC1 | na | 16639 | 0.012 | 0.1493 | Yes |
| 52 | ATP6V0D1 | na | 16641 | 0.012 | 0.1527 | Yes |
| 53 | NDUFS3 | na | 16717 | 0.011 | 0.1538 | Yes |
| 54 | UQCRC2 | na | 16785 | 0.011 | 0.1551 | Yes |
| 55 | COX4I1 | na | 16804 | 0.011 | 0.1577 | Yes |
| 56 | ATP6V1F | na | 16959 | 0.010 | 0.1561 | Yes |
| 57 | COX7C | na | 17085 | 0.009 | 0.1551 | Yes |
| 58 | NDUFS7 | na | 17112 | 0.009 | 0.1569 | Yes |
| 59 | NDUFA4 | na | 17126 | 0.009 | 0.1590 | Yes |
| 60 | NDUFB9 | na | 17132 | 0.009 | 0.1614 | Yes |
| 61 | UQCRQ | na | 17160 | 0.008 | 0.1631 | Yes |
| 62 | COX6C | na | 17183 | 0.008 | 0.1649 | Yes |
| 63 | NDUFA3 | na | 17211 | 0.008 | 0.1664 | Yes |
| 64 | COX17 | na | 17227 | 0.008 | 0.1683 | Yes |
| 65 | NDUFA5 | na | 17695 | 0.005 | 0.1563 | No |
| 66 | COX5A | na | 17706 | 0.005 | 0.1575 | No |
| 67 | CYC1 | na | 17708 | 0.005 | 0.1589 | No |
| 68 | ATP6V1C1 | na | 17751 | 0.005 | 0.1590 | No |
| 69 | NDUFV1 | na | 17902 | 0.003 | 0.1557 | No |
| 70 | NDUFV2 | na | 18101 | 0.002 | 0.1506 | No |
| 71 | UQCRH | na | 18129 | 0.002 | 0.1503 | No |
| 72 | SDHB | na | 18300 | 0.001 | 0.1456 | No |
| 73 | ATP6V0A1 | na | 18461 | -0.001 | 0.1411 | No |
| 74 | NDUFS5 | na | 18515 | -0.001 | 0.1399 | No |
| 75 | ATP6V1D | na | 18807 | -0.003 | 0.1324 | No |
| 76 | PPA1 | na | 18823 | -0.003 | 0.1328 | No |
| 77 | NDUFA2 | na | 18897 | -0.004 | 0.1318 | No |
| 78 | COX6A1 | na | 18979 | -0.004 | 0.1307 | No |
| 79 | ATP6V1A | na | 19008 | -0.005 | 0.1313 | No |
| 80 | NDUFB4 | na | 19246 | -0.006 | 0.1263 | No |
| 81 | UQCRFS1 | na | 19283 | -0.007 | 0.1273 | No |
| 82 | UQCRB | na | 19342 | -0.007 | 0.1278 | No |
| 83 | ATP6V1G1 | na | 19379 | -0.008 | 0.1289 | No |
| 84 | ATP6V0C | na | 19779 | -0.011 | 0.1207 | No |
| 85 | ATP6V1E1 | na | 20236 | -0.015 | 0.1120 | No |
| 86 | NDUFA6 | na | 20241 | -0.015 | 0.1163 | No |
| 87 | ATP6V1C2 | na | 20822 | -0.020 | 0.1056 | No |
| 88 | ATP6V1H | na | 21127 | -0.023 | 0.1036 | No |
| 89 | COX7A1 | na | 21665 | -0.029 | 0.0965 | No |
| 90 | NDUFB6 | na | 21907 | -0.031 | 0.0987 | No |
| 91 | SDHA | na | 22031 | -0.032 | 0.1047 | No |
| 92 | COX10 | na | 22102 | -0.033 | 0.1124 | No |
| 93 | ATP6V1E2 | na | 24858 | -0.069 | 0.0531 | No |
| 94 | ATP12A | na | 26906 | -0.106 | 0.0251 | No |
| 95 | ATP6V1B1 | na | 27614 | -0.121 | 0.0404 | No |
| 96 | ATP6V1G2 | na | 27824 | -0.127 | 0.0718 | No |
| 97 | COX8C | na | 27951 | -0.130 | 0.1064 | No |
| 98 | ATP4A | na | 32635 | -0.303 | 0.0604 | No |
Table: GSEA details [plain text format]

  

Fig 2: KEGG\_OXIDATIVE\_PHOSPHORYLATION      
 Blue-Pink O' Gram in the Space of the Analyzed GeneSet

  

Fig 3: KEGG\_OXIDATIVE\_PHOSPHORYLATION: Random ES distribution      
 Gene set null distribution of ES for **KEGG\_OXIDATIVE\_PHOSPHORYLATION**
